# Are Cement Spacers and Beads Loaded with the Correct Antibiotic(s) at the Site of Periprosthetic Hip and Knee Joint Infections?

**DOI:** 10.3390/antibiotics10020143

**Published:** 2021-02-01

**Authors:** Konstantinos Anagnostakos, Ismail Sahan

**Affiliations:** Zentrum für Orthopädie und Unfallchirurgie, Klinikum Saarbrücken, D-66119 Saarbrücken, Germany; ssahan@klinikum-saarbruecken.de

**Keywords:** hip spacer, knee spacer, antibiotic-loaded bone cement, antibiotic-impregnated bone cement, infection persistence, reinfection

## Abstract

The optimal impregnation of antibiotic-loaded bone cement in the treatment of periprosthetic hip and knee joint infection is unknown. It is also unclear, whether a suboptimal impregnation might be associated with a higher persistence of infection. A total of 93 patients (44 knee, 49 hip) were retrospectively evaluated, and the most common organism was a methicillin-resistant *Staphylococcus epidermidis*, followed by methicillin-susceptible *Staphylococcus aureus*. Of all the organisms, 37.1% were resistant against gentamicin and 54.2% against clindamycin. All organisms were susceptible against vancomycin. In 41 cases, gentamicin-loaded beads were inserted and in 52 cases, spacers: (2 loaded only with gentamicin, 18 with gentamicin + vancomycin, 19 with gentamicin + clindamycin, and 13 with gentamicin + vancomycin + clindamycin). The analysis of each antibiotic impregnation showed that complete susceptibility was present in 38.7% of the cases and partial susceptibility in 28%. In the remaining 33.3%, no precise statement can be made because either there was a culture-negative infection or the antibiotic(s) were not tested against the specific organism. At a mean follow-up of 27.9 months, treatment failure was observed in 6.7% of the cases. Independent of which antibiotic impregnation was used, when the organism was susceptible against the locally inserted antibiotics or not tested, reinfection or persistence of infection was observed in the great majority of cases. Future studies about the investigation of the optimal impregnation of antibiotic-loaded bone cement are welcome.

## 1. Introduction

Periprosthetic hip and knee joint infections (PJI) pose a rare but hazardous complication. At the site of late infections, a two-stage protocol is considered to be the treatment of choice in Europe and North America [1,2], whereas single reports demonstrate equally good results for the one-stage exchange arthroplasty [3,4]. Independent of which surgical strategy is applied, it is universally accepted that the success of each procedure is based on three columns: the surgical debridement of all infected, necrotic and ischemic tissue including removal of all affected prosthetic components; as well as local and systemic antibiotic therapy. With regard to the local antibiotic therapy, antibiotic-loaded acrylic bone cement (ALAC) is established to be a valuable device although no prospective study has yet demonstrated its efficacy over systemic antibiotics alone [5]. Either in the form of beads or spacers, ALAC provides high local antibiotic concentrations during the postoperative period, which vastly exceeds those after systemic administration, and has low or no systemic side effects [1].

Depending on the particular causative organism (if preoperatively known) and its antibiotic resistance profile, commercially available ALAC can be additionally loaded with various antibiotics. Literature data show that in most cases ALAC is impregnated with a combination of an aminoglycoside and a glycopeptide because it produces a broader antimicrobial spectrum and has a synergistic effect on their pharmacokinetic properties [1]. Industrial pre-fabricated ALAC is available with the impregnation of a single antibiotic (aminoglycoside) or an antibiotic combination (aminoglycoside + glycopeptide or aminoglycoside + lincosamide) [6].

Clinical practice shows that the identification of the causative bacterium does not always succeed preoperatively. Moreover, even in cases with a positive preoperative microbiologic result, the examination of the intraoperatively taken tissue samples might demonstrate the presence of additional bacteria. Therefore, it cannot always be guaranteed that ALAC will be loaded with the correct antibiotic(s). However, an inappropriate or a suboptimal impregnation of cement beads or spacers might have a negative impact on the clinical course and the eradication of the joint infection. Should any bacteria that are resistant against the locally placed antibiotic agents survive the surgical debridement, the risk of persistent infection might dramatically rise [7]. Other concerns include the well-known limitations of commonly used antibiotics, ineffectiveness on bacteria within biofilms, bacterial colonization of ALAC after the antibiotic release decreases to subtherapeutic levels and alterations to the host microbiome [8].

To the best of our knowledge, no study has tried to investigate in a large collective whether cement beads and spacers are loaded with the “correct” antibiotic(s), and what impact this might have on the infection eradication rate at the site of periprosthetic hip and knee joint infections.

## 2. Results

### 2.1. Demographic Data

A total of 93 consecutive patients (46 men, 47 women, mean age 70.6 (35–88) y.) were included into the study. There were 44 knee (6 articulating, 38 static spacers) and 49 hip cases (8 spacer, 41 Girdlestone). All demographic data are summarized in Table 1.

### 2.2. Microbiological Findings

In 71 of the 93 cases (76.3%), at least one pathogenic organism could be identified (57 mono-and 14 polymicrobial infections). In the remaining 22 cases, the microbiological examination revealed no bacteria growth (“culture-negative”); however, the histopathological findings confirmed the presence of an active infection in each case. In 57 of the 93 cases (61.3%) either an open biopsy or aspiration of joint fluid was preoperatively carried out, revealing positive results in 89.5% of the cases (51/57).

A total of 90 different organisms (77 Gram-positive, 11 Gram-negative) were identified (Table 2). In 2 cases, a fungal infection was present. The most common organism was a methicillin-resistant *Staphylococcus epidermidis* (MRSE) in 23 cases, followed by methicillin-susceptible *Staphylococcus aureus* (MSSA) and *beta-haemolytic streptococci* in 13 and 8 cases, respectively.

**Figure 1:**
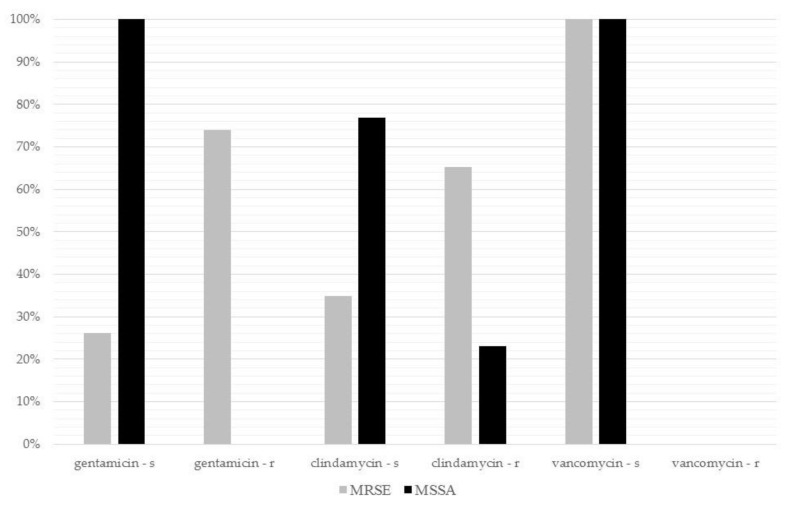
Comparison of the antibiotic resistance rates of methicillin-resistant *S. epidermidis* (MRSE) and methicillin-susceptible *S. aureus* (MSSA) against gentamicin, clindamycin and vancomycin.

Among the bacteria tested for susceptibility against gentamicin, 62.9% were susceptible and 37.1% resistant. Regarding clindamycin, 45.8% of the bacteria were susceptible and 54.2% resistant. No organism demonstrated a resistance against vancomycin (100% susceptibility). Due to the small sample size of various identified organisms, the specific resistance profile was analyzed only for MRSE and MSSA (Figure 1). The resistance rates of MRSE against gentamicin and clindamycin were higher compared with those of MSSA, respectively (Figure 1).

### 2.3. Antibiotic Impregnation of the Spacers/Beads and Resistance Rates

**Figure 2:**
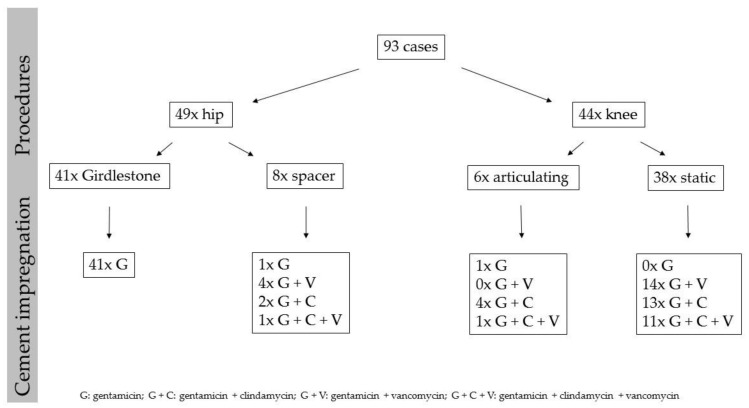
Data about the cement impregnation of spacers and beads at the site of 93 periprosthetic hip and knee joint infections.

All beads were solely loaded with gentamicin. Among the 52 spacers evaluated for this study, 2 were loaded with gentamicin, 18 with gentamicin + vancomycin, 19 with gentamicin + clindamycin, and 13 with gentamicin + vancomycin + clindamycin. All data about the cement impregnation of the spacers in the whole collective as well as the particular subgroups and the beads is shown in Figure 2. 

The analysis of each antibiotic impregnation with regard to complete or partial susceptibility against the particular pathogen organism(s) showed that a complete susceptibility was present in 38.7% of the cases (36/93) and a partial susceptibility in 28% (26/93). In the remaining 33.3% of the cases (31/93) no precise statement can be made because either there was a culture-negative infection or the antibiotic(s) were not tested against the specific organism (e.g., no testing of Gram-negative organisms against vancomycin). All details about each antibiotic impregnation in each group are summarized in Table 3.

### 2.4. Clinical and Infection Outcome 

From the 93 included cases, 9 patients were lost in the follow-up, and 8 passed away for reasons not related to their infection. Among the remaining 76 patients, 60 (27x hip, 33x knee) had a follow-up of at least 1 year (mean 27.9 months; min–max 12–44 months) and could be evaluated.

In the hip group, 21 patients underwent prosthesis reimplantation after a mean interim phase of 59.6 days. Six patients permanently retained a resection arthroplasty as a definitive solution because either their comorbidities did not allow for a later prosthesis reimplantation or the patients refused the second surgery. At a mean follow-up of 28 months (min–max 12–41 months) one patient showed persistence of infection, and three patients suffered from reinfection. 

**Figure 3:**
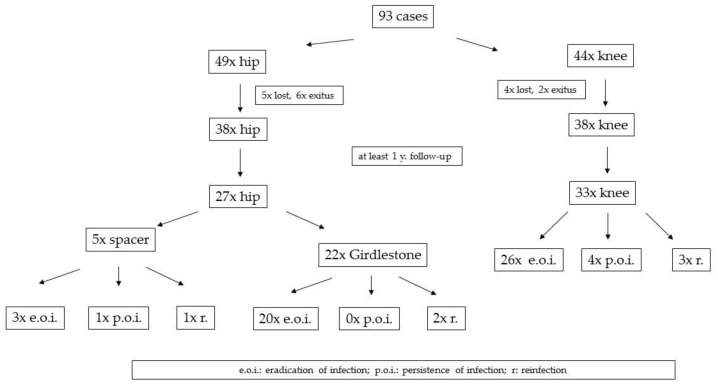
Infection outcome.

In the knee group, 31 patients had prosthesis reimplantation, and two patients were treated by a nail arthrodesis (mean interim phase 68.7 days). At a mean follow-up of 27.6 months (min–max 12–44 months), three patients showed persistence of infection, and four patients suffered from reinfection. In the whole collective, treatment failure was observed in 6.7% of the cases (Figure 3). All cases with a persistence of infection occurred between 6 months and 4 years after the second stage, with most of them taking place within the first 12 months. All patients were treated again with a two-stage procedure and had no further complications during the next follow-up.

Independent of which antibiotic impregnation was used, when the organism was susceptible against the locally inserted antibiotics or not tested, reinfection or persistence of infection was observed in the great majority of cases. (Table 3). The most cases of reinfection and persistence of infection, respectively were seen, when gentamicin-vancomycin-loaded spacers were implanted.

## 3. Discussion

The management of periprosthetic hip and knee joint infections can be challenging. At the site of a two-stage procedure, ALAC plays a central role in the eradication of the infection. To guarantee the correct impregnation of bone cement, two things are essential: the preoperative identification of the causative pathogen organism, in order to know against which antibiotics the particular organism is or is not susceptible, and adequate knowledge about the pharmacokinetic properties of ALAC itself. 

The preoperative diagnostic measures in revision arthroplasty surgery are still a topic of debate. Several criteria have been proposed by various societies, such as the Musculoskeletal Infection Society [9], the International Consensus Meeting on Periprosthetic Joint Infection [10], and the European Bone and Joint Infection Society [11]. However, none of them has been universally accepted yet. A reliable and valid pre- and intra-operative diagnostic tool with 100% specificity and 100% sensitivity in the diagnosis or exclusion of a PJI is still lacking [12]. 

Based on this problem, several studies have focused on this topic in the past years. However there still exists no tool or biomarker that is regarded to be the gold standard. Although a single abnormality in inflammation parameters, such as the erythrocyte sedimentation rate (ESR) or the C-reactive protein (CRP) value, has been reported to increase the likelihood of both infection or reoperation following revision arthroplasty [13], an elevation of the CRP values could also be attributed to other causes like cardiovascular, gastrointestinal, urologic or respiratory problems or even unknown causes [14]. On the other side, normal CRP and white blood cell count values cannot rule out a PJI [15]. Synovial biomarkers might play a role in the future, but they are currently not established in a clinical setting. The analysis of antimicrobial peptides and proinflammatory cytokines might provide valuable information for the diagnosis of PJI [16]. Other authors described an increase in interleukins such as IL-1 and IL-6 in synovial fluid at the site of PJIs [17,18]. The use of the synovial alpha 1 defensin [19,20] and the synovial leucocyte esterase strip tests [12,21] certainly point to an improvement for the intraoperative diagnosis. However, disadvantages for both tests are well known (e.g., the costs of the rapid lateral flow test for the former and the possibility that blood in the synovial fluid could interfere with the color change of the urinalysis strip for the latter) [19]. Nuclear imaging techniques, such as leucocyte scintigraphy, have a sensitivity and specificity of 88% and 92%, respectively [22] but are not routinely performed and depend on the particular surgical indication. The use of the sonication has demonstrated promising results in several studies, whereas controversy still exists regarding the universal use of this technique [23], and this method does not help in the pre- and intra-operative setting.

In more than 50% of the cases in the present study, the causative organism was preoperatively known. The microbiological examination showed that staphylococci were the most common identified organisms, which is consistent with other literature data [24,25]. Of all organisms, 37.1% were resistant against gentamicin and 54.2% against clindamycin. All were susceptible against vancomycin. Interestingly, a relatively high incidence rate of culture-negative PJI with 23.6% was observed, which is higher than the other rates reported in the literature [26]. We cannot interpret this finding with certainty. Although further microbiological methods, such as a broad-range 16S rRNA polymerase chain reaction, have been additionally carried out in certain cases beside the standard cultures, this high rate emphasizes the necessity for the optimization of the microbiological detection methods in the future.

Knowledge of the pharmacokinetic properties of ALAC is an indispensable element in the successful treatment of PJI. It is known that not every antibiotic equally qualifies for incorporation into bone cement. Desirable characteristics include availability in powder form, thermal stability, low influence on the mechanical properties of the bone cement, elution in high concentrations for prolonged periods, a wide antimicrobial spectrum, and being bactericidal in low concentrations [27]. Moreover, it is accepted that industrially fabricated ALAC provides a more homogenous antibiotic elution than does ALAC that has been manually loaded with additional antibiotics during surgery [28]. Last but not least, the combination of different antibiotic groups (mostly an aminoglycoside with a glycopeptide) demonstrates a synergistic effect with the amount of the released antibiotic amounts as well as with the level above the minimal inhibitory concentration of the particular causative organism, whereas this effect strongly depends on the antibiotic ratio used for impregnation of the bone cement [27,28]. In addition to that, the efficacy of ALAC can be also be thwarted by some additional mechanisms. Interstitial fluid flow from the wound to the peripheral tissue creates a convection current that drives released antibiotic amounts away from the bacteria [29]. Moreover, in an animal model, *S. aureus* was able to deform, proliferate, and migrate into the osteocyte lacuno-canalicular network, thus making it extremely difficult for the locally released antibiotics to be effective [30].

Despite numerous studies on this topic, hard scientific data about the optimal antibiotic impregnation of bone cement in the management of PJI is missing. Data from the literature provide only scarce recommendations that are mostly based on personal experiences [27] or published after a consensus meeting [31]. On the other side, concerns have been expressed that, when bone cement has been impregnated with the “wrong” antibiotics or the antibiotic elution has decreased over time, spacers might act as foreign bodies that could again be colonized by bacteria and support the persistence of infection [32,33,34,35]. Despite the presence of single studies reporting on bacterial growth on antibiotic-loaded spacers, these literature data should be critically evaluated. In some cases, the spacers have not been loaded with antibiotics at all [32], so it is not surprising that these spacers demonstrated bacterial growth on their surface.

The correct impregnation of the bone cement remains a great challenge even if all the aforementioned information is taken into consideration. Although in more than half of the cases, the causative organism was preoperatively known: complete susceptibility against each particular impregnation was present in 38.7% and a partial one in 28% of the cases. The identification of additional organisms to the one(s) preoperatively detected or a change in the planned procedure (e.g., Girdlestone arthroplasty instead of spacer implantation due to intraoperative femoral fracture or unexpected transfemoral approach) are factors that cannot be preoperatively excluded and are frequently met in those revision surgeries. Therefore, to expect to implant 100% correctly impregnated spacers or beads is not realistic. In the present study, 4 different impregnations were used, which represents the necessity for individuality of treatment at the PJI site. Unfortunately, due to the retrospective study design and the involvement of several surgeons in the management of these cases, we cannot identify the precise criteria according to which the particular spacer was loaded in each individual case.

Using a sole interpretation of the resistance profile as a basis for the cement impregnation might also mislead the treating surgeon regarding the ideal antibiotic choice. Based on the numbers of the antibiotic resistance determined in the present study, it might appear at first sight always advisable to impregnate bone cement with vancomycin since no organism demonstrated a resistance against it. On the other hand, it could have been expected that clindamycin-containing spacers might had been more frequently involved in cases with infection persistence. The results of the present study could support neither the first nor the second hypothesis. 

There are several possible explanations for these observations. The sample size in each group might have been too small to justify such expectations and certainly deserves further future investigation. The antibiotic impregnation itself (industrial vs. manual) could be also another reason. Another possible explanation for not identifying suboptimal cement impregnation as a sole risk factor for treatment failure is the fact that the surgical debridement and the postoperative systemic antibiotic therapy also play a role in the eradication of the infection. A major advantage of the two-stage procedure compared with the one-stage procedure is that the surgeon has the opportunity to debride twice, not only once, thus optimizing the bacteria load reduction. The treatment failure rate of the present study was 6.7% at a mean follow-up of 27.9 months, which is at least similar to the results of other studies [36,37,38]. Interestingly, the great majority of the cases that had to be revised during the follow-up occurred within the first year. This emphasizes the importance of a narrow monitoring of these patients during follow-up, especially within the first 12 months.

Our study has certainly some limitations. This is a retrospective work with all drawbacks of such a study design. A valid or even statistical analysis of the present data is difficult to perform, since several surgeons were involved in the treatment of the cases, and there are several types of spacers with a partly low number of cases. The present results can be only interpreted within this descriptive study. Nevertheless, this is the first study that tried to investigate this topic, and these results might act as a basis for future studies. 

## 4. Materials and Methods 

All patients, who were treated in our department between 2016 and 2020 with a two-stage exchange arthroplasty at the site of late periprosthetic hip and knee joint infections, were regarded to be potential candidates for inclusion in the study. The patients’ records were retrospectively evaluated with regard to the following parameters: demographic data (age and gender), microbiological and histopathological findings, type of ALAC device used (beads, spacers), type of cement impregnation, joint infection outcome, and length of follow-up.

All patients suffering from a late periprosthetic hip or knee infection were treated according to an identical algorithm in our department. The infection was defined by the criteria of the Musculoskeletal Infection Society (MSIS) [9]. Preoperatively, a joint aspiration was performed to differentiate aseptic from septic prosthesis loosening, except for those patients whose positive blood cultures confirmed hematogenous infections or those who came with systemic sepsis signs and were immediately operated on. A further exemption concerned patients who had fistulas. In these cases, we preferred to take direct tissue samples during surgery. If joint aspiration revealed negative microbiological findings, but clinical, laboratory or radiological findings pointed strongly to the presence of an infection, an arthroscopic or open biopsy was performed prior to the prosthesis revision.

All cases were treated in a two-stage procedure. In the first surgery, all prosthetic components including cement were removed, and all infected, necrotic or ischemic tissue layers were debrided. A pulsatile lavage with at least 5 L Ringer’s solution was always performed. Tissue samples from at least 5 different locations along with joint fluid were taken and sent for further microbiological and histological examination. Until 2018, all samples were cultured over 7 days. Since 2019, the culture period was extended to 14 days because some bacteria can be only detected after a prolonged culture [39]. Regarding the histological findings, all samples were classified in accordance with the system of Krenn and Morawietz [40]. For cases with negative culture but positive histological findings, the samples were further investigated by means of a broad-range 16S rRNA polymerase chain reaction.

At the site of hip infections, the primary goal has always been to implant an antibiotic-loaded spacer. In these cases, the spacer was intraoperatively produced by means of commercially available moulds (Stage One^TM^, Fa. ZimmerBiomet, Freiburg im Breisgau, Germany). However, patients with a reduced medical condition and unable to avoid putting any weight on the operated extremity postoperatively, those who suffered from large osseous defects of the proximal femur or acetabulum and those who needed a transfemoral approach for the safe removal of the femoral stem, were deemed better suited for a resection arthroplasty (Girdlestone procedure) due to the higher theoretical risk of a secondary spacer dislocation or fracture during the interim phase [41]. In these cases, 2–3 antibiotic-loaded beads (Septopal^®^, Fa. ZimmerBiomet, Freiburg im Breisgau, Germany) were inserted into the acetabulum and the femoral canal. 

Regarding knee infections, the presence of bone defects, according to the Anderson Orthopedic Research Institute (AORI) bone defect protocol [42], helped us decide, whether an articulating or a static spacer should be implanted. All patients with bone defects I-IIA were treated with an articulating spacer (Copal knee moulds, Fa. Hereaus, Wehrheim, Germany). Patients suffering from bone defects IIB-III were treated with a static spacer. This spacer was moulded individually according to the particular joint space geometry. 

For the intraoperative production of hip and knee spacers, commercially available antibiotic-loaded bone cement was used, which was loaded either with gentamicin or gentamicin + clindamycin (Palacos^®^ R+G/Copal^®^ G+C, Fa. Hereaus, Wehrheim, Germany). Depending on the particular causative organism and its resistance profile, 2 g vancomycin/40 g bone cement were additionally incorporated into the cement in certain cases. 

After the operation, an immediate, systemic antibiotic therapy was started; either specific, if the causative organism was preoperatively known, or a calculated therapy with 1.5 g cefuroxime intravenously, if the causative organism were unknown, and adjusted if necessary during the further course. All patients received an antibiotic therapy over 6 weeks, consisting of 3–4 weeks intravenously and 2–3 weeks orally. All knee joints with a static spacer were immobilized in a cast in full extension. Patients with an articulating spacer were allowed to flex their knee as tolerated. All patients (hip and knee) were allowed to walk on crutches with no weight on the operated extremity.

**Figure 4:**
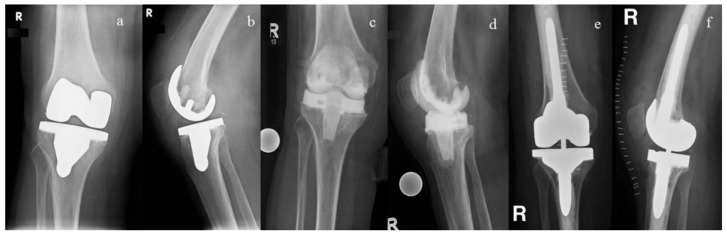
(**a**,**b**) Anterio-posterior (**a**) and lateral (**b**) radiographs of the right knee of a 69-year-old woman with a septic loosening of the tibial component; (**c**,**d**) After prosthesis explantation, an articulating spacer was implanted; (**e**,**f**) Following an interim period of 54 days, a condylar-constrained prosthesis was implanted (Triathlon TS^®^, Fa. Stryker, Duisburg, Germany).

**Figure 5:**
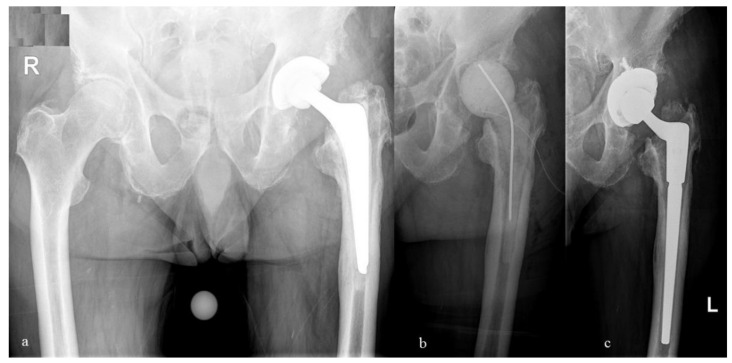
(**a**) Anterio-posterior radiographs of the pelvis of an 86-year-old man with a septic loosening of the femoral stem; (**b**) Following prosthesis explantation, an articulating spacer was inserted into the femur; (**c**) After an interim period of 69 days, the prosthesis reimplantation was performed with cementless implants (Restoration Acetabular Shell^®^, Restoration Stem^®^, Fa. Stryker, Duisburg, Germany).

**Figure 6:**
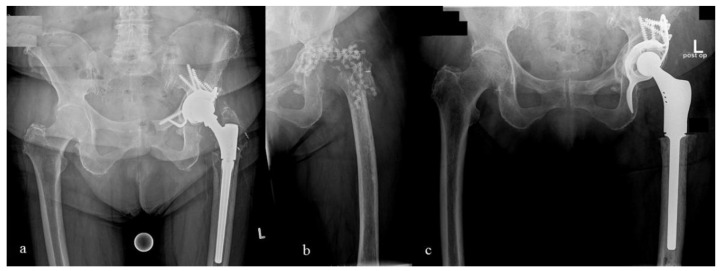
(**a**) Anterio-posterior radiographs of the pelvis of 78-year-old woman. Notice that anchors are already present in the major trochanter indicating a prior refixation; (**b**) During explantation, a refracture of the extremely weakened major trochanter occurred, so a resection arthroplasty was carried out instead of a spacer implantation; (**c**) Following an interim period of 54 days, the prosthesis reimplantation was performed (Burch-Schneider^®^ antiprotrusio cage, cemented polyethylene cup, Fa. ZimmerBiomet, Freiburg im Breisgau, Germany; GMRS^®^, Fa. Stryker, Duisburg, Germany).

Six weeks after the spacer implantation or the Girdlestone procedure, the antibiotic therapy was paused for 7–10 days and the serum inflammation parameters (C-reactive protein, blood cell count) controlled. If the laboratory parameters were normal, a prosthesis reimplantation was then planned if the wound had healed and the general medical condition of the patient allowed for it. The type of implants used were chosen based on the amount of bone loss and quality (Figure 4, Figure 5 and Figure 6). A joint aspiration was not routinely carried out prior to spacer explantation and prosthesis reimplantation because the literature data has demonstrated no benefit to such a measure [43,44].

At reimplantation, soft-tissue specimens were taken again and sent for microbiological and histopathological examination. In the event of the macroscopical presence of pus or other tissue signs that might have been suspicious for the persistence of infection, the joint was debrided again and the spacer only exchanged. All patients who underwent prosthesis reimplantation did not receive postoperatively any systemic antibiotic therapy.

“Persistence of infection” pertained to those cases that showed infection by the same pathogen organism that was first identified. “Reinfection” pertained to those cases that suffered from infection by a different organism than was first detected. “Treatment failure” was defined only by the persistence of infection.

The study was conducted in accordance with the *Declaration of Helsinki*.

## 5. Conclusions

The management of hip and knee PJI is a complex procedure. The ideal impregnation of bone cement for the management of these infections by means of spacers or beads is still unknown. The present study could show a high rate of resistance among the causative organisms against gentamicin and clindamycin, which however did not lead directly to treatment failure. Although it is not based on hard scientific data, the authors recommend impregnating spacers with all three antibiotics (gentamicin + clindamycin + vancomycin) in order to achieve the best possible antimicrobial effect until the ideal cement impregnation is defined. Future studies are required in order to enhance the antibiotic impregnation of bone cement, and hence help optimize the treatment of periprosthetic hip and knee joint infections.

## Figures and Tables

**Table 1:** Demographic data of all included patients.

	n =	Gender	Mean Age (Years) (Min–Max)
total collective	93	46 male, 47 female	70.6 (35–88)
hip (total)	49	19 male, 30 female	72.1 (35–88)
hip (Girdlestone)	41	13 male, 28 female	72.8 (35–88)
hip (spacer)	8	6 male, 2 female	68.6 (42–86)
knee (total)	44	27 male, 17 female	69.1 (51–87)

**Table 2:** Microbiological findings.

Pathogen Organism	n =
MRSE	23
MSSA	13
*beta-hem. streptococci*	8
*E. faecalis*	7
*E. faecium*	5
MSSE	4
*C. acnes*	4
*S. caprae*	3
*P. micra*	2
*E. coli*	2
*S. hemolyticus*	2
*Ps. aeruginosa*	2
MRSA	1
*C. koseri/diversus*	1
*S. warneri*	1
*Str. gallolyticus*	1
*Enterobacteriacae*	1
*Viridans streptococci*	1
*Streptococci*—n.f.d.	1
*S. hominis*	1
*S. marcescens*	1
*C. albicans*	1
*P. guillermondii*	1
*P. mirabilis*	1
*E. cloacae*	1
*M. morganii*	1
*Veilonella parvula/tobetsuensis*	1
negative	22

MRSE: methicillin-resistant *Staphylococcus epidermidis*; MSSA: methicillin-susceptible *Staphylococcus aureus*; MSSE: methicillin-susceptible *Staphylococcus epidermidis*; MRSA: methicillin-resistant *Staphylococcus aureus*; n.f.d.: no further differentiation.

**Table 3:** Evaluation of each antibiotic impregnation with regard to the susceptibility of the particular pathogen organism(s) and the emergence of persistence of infection or reinfection.

		Hip Girdlestone (n = 41)	Hip Spacer (n = 8)	Knee Spacer (n = 44)	Total Collective (n = 93)	Persistence of Infection	Reinfection
G	s	20	0	0	20	0	2
	p.s.	9	0	0	9	0	0
	n.t./n.r.	12	1	1	14	0	0
G + C	s	0	0	5	5	1	0
	p.s.	0	2	7	9	0	0
	n.t./n.r.	0	0	5	5	0	0
G + V	s	0	1	7	8	2	1
	p.s.	0	1	6	7	0	0
	n.t./n.r.	0	2	1	3	0	3
G + C + V	s	0	0	3	3	1	0
	p.s.	0	1	4	5	0	0
	n.t./n.r.	0	0	5	5	0	1

G: gentamicin; G + C: gentamicin + clindamycin; G + V: gentamicin + vancomycin; G + C + V: gentamicin + clindamycin + vancomycin; s: susceptible; p.s.: partly susceptible; n.t./n.r.: not tested/not relevant.

## Data Availability

The data presented in this study are available on request from the corresponding author.
